# Sustainability Competencies in Higher Education Research: An Analysis of Doctoral Theses in Portugal

**DOI:** 10.3390/ejihpe12040028

**Published:** 2022-03-28

**Authors:** Patrícia Sá, Mónica Lourenço, Vânia Carlos

**Affiliations:** Research Centre for Didactics and Technology in the Education of Trainers (CIDTFF), Campus Universitário de Santiago, University of Aveiro, 3810-193 Aveiro, Portugal; monicalourenco@ua.pt (M.L.); vania.carlos@ua.pt (V.C.)

**Keywords:** education for sustainability, key competencies in sustainability, Portuguese higher education, doctoral theses

## Abstract

Educational research has been highlighting the importance of defining key competencies and learning outcomes related to education for sustainability as a reference for the transparent evaluation of students’ learning in this domain. Drawing on a reference framework that identifies five key competencies in sustainability (i.e., systems-thinking competency, anticipatory competency, normative competency, strategic competency, and interpersonal competency), the study reported in this paper aims to understand whether, how, and to what extent these competencies are present in doctoral theses in Higher Education published in Portugal in the past ten years. To address this objective, a qualitative study framed in an interpretative paradigm was conducted, and a literature review was used as a preferential research method to elicit meaning, gain understanding, and develop empirical knowledge. The retrieved documents were treated using deductive content analysis, which was performed using WebQDA software. Results of the analysis show that the competencies considered in the reference framework are present in research on education for sustainability carried out in recent years in Portugal, with a greater emphasis on strategic and anticipatory competencies. Findings suggest that it is important to continue to conduct research on these competencies to successfully integrate them into educational curricula and teacher education programs.

## 1. Introduction

The first two decades of the 21st century have been marked by a series of interconnected economic, social, political, and environmental crises that have affected communities and individuals across the globe: Terrorism and extremist violence, the financial crash, natural disasters, mass migration and refugee waves, gender and racial discrimination, the rise of populism, and, more recently, a deadly virus are but some examples of the “bigger-than-self” challenges facing society. Humanity is, therefore, at a crossroads regarding the legacy we wish to leave for future generations. On the one side lies the continuous expansion of democracy, the defense of human rights and freedoms, and concerted efforts to address inequalities of all sorts, as well as the present and growing threats of global climate change; on the other side lies the dismantling of democracy in lieu of populist and authoritarian regimes, increased attacks on the marginalized and more vulnerable populations of the world, and acceleration in the degradation of Planet Earth.

In this context, sustainability appears as a “real utopia” [1], a possible dream to help us rethink our relationships with others and nature and guide us on the path towards transformative change. The Sustainable Development Goals (SDGs), a collection of 17 interdependent goals included in the 2030 Agenda, which were designed to be a blueprint to achieve a better and more sustainable future, are a real utopia, a general orientation towards a better society providing a sense of direction for societal change [2]. Despite their contradictions and fallacies [3], the SDGs represent an unparalleled effort and a commitment towards social justice for all and for the planet, perhaps only matched by the Human Rights Declaration of the 20th century. Within the SDGs, Goal 4 on quality education remains the most critical and ambitious goal, foregrounding the role of education in building societies that are more peaceful and sustainable. Target 4.7., in particular, states:

“By 2030, ensure that all learners acquire the knowledge and skills needed to promote sustainable development including, among others, through education for sustainable development and sustainable lifestyles, human rights, gender equality, promotion of a culture of peace and non-violence, global citizenship and appreciation of cultural diversity and of culture’s contribution to sustainable development”.

[4]

Education is, then, key to the realization of the 2030 Agenda. Indeed, the four areas of sustainability—economic growth, social inclusion, governance, and environmental protection—depend on the contribution of citizens that are informed about the world around them and engage actively in their own communities. This places SGD 4.7. at “the very heart of the sustainability agenda” ([5], p. 808).

Solving sustainable development complex problems requires, among other things, citizenship education, holistic and transdisciplinary teaching and learning approaches, and a values-based education oriented towards the promotion of critical thinking skills and troubleshooting [6]. Moreover, it is fundamental to empower future citizens as informed decision makers, capable of valuing distributive justice, and with skills to make choices oriented towards the future, committing to a socially just and peacefully developed world. Therefore, there is growing interest among researchers and practitioners in developing participatory citizenship attitudes and democratic values, especially in children and young people, so that they can build their own social and political identities, make informed decisions, and participate actively in the day-to-day life of their own communities [7]. Thus, besides being at the center stage of political decision making, citizenship must also be perceived as a practice of someone playing an active role in their own community [8].

In the midst of this urgency emerges the relevance of educating for Sustainable Development, whose guidelines imply a profound change in the design and implementation of strategies for teaching and learning that serve this purpose, anchored in active learning methodologies and supported by adequate resources [9]. The literature [10,11,12] also stresses the importance of defining key competencies and learning outcomes related to sustainability education, as a reference for a transparent evaluation of students’ learning in this domain. The UNESCO [13] also recognizes its relevance in the report “Education for sustainable development goals: Learning objectives”.

Sustainability competencies are educational concepts focused on asking what problem-solving strategies, concepts, and abilities for social action should be developed in learners, which are not restricted by subject boundaries or specific content knowledge [14]. The academic report “Framework for education for sustainability: enhancing competences in education” [15] proposes the sustainability competencies described below:

- Systems-thinking competency aims to develop the ability to address sustainability problems from a wider and more holistic perspective, focusing on understanding the intermediate and root causes of complex problem constellations;
- Anticipatory competency involves specific analysis skills that focus on possible future trajectories and scenarios (sustainability issues, sustainability problem-solving frameworks, imagination play, creativity);
- Normative competency is the ability to map, specify, apply, reconcile, and negotiate values, principles, goals, and targets;
- Strategic competency is the ability to design and implement strategic plans towards sustainability that could justifiably avoid undesirable scenarios (interventions, transitions, transformative governance strategies);
- Interpersonal competency is closely connected to learning and implementing all other sustainability competencies, implying social knowledge and skills (communicating, deliberating, negotiating, collaborating, leadership, pluralistic and trans-cultural thinking, and empathy).

Table 1 presents the framework with the five components, as well as the corresponding analysis features for each component.

Anchored on Wiek, Withycombe, and Redman [9], this study uses the framework described in Table 1 as a matrix to identify the key competencies in sustainability that are present in doctoral theses in Education published in Portugal over the past ten years. In particular, we use a literature review supported by deductive content analysis to understand whether, how, and to what extent these key competencies are present in these documents. Considering the current context and international calls to integrate sustainability issues into curricula worldwide, we feel that this is a timely study with valuable implications for educational practice and assessment, as well as for teacher education.

## 2. Methodology

### 2.1. Context and Aims of the Study

This study was developed within the Erasmus+ project “TEDS—Teacher education for sustainability. Schools educating for sustainability: proposals for and from in-service teacher education” (project code: 2019-1-PT01-KA201-060830). TEDS is a three-year collaborative project coordinated by the University of Aveiro (Portugal), which started in 2019. The project involves a network of researchers, teacher educators, and teachers from four other European universities—the University of Helsinki (Finland), the University of Malta (Malta), Vytautas Magnus University (Lithuania), and the University of Nantes (France)—and counts on the collaboration of teacher education centers and schools in all of these countries.

TEDS main goal is to contribute to teacher education for sustainability in Europe considering the following dimensions: Equity and social solidarity; diversity, dialogue, and inclusion; natural resources, environment, and technology; and economic and financial literacy. This goal is to be achieved by promoting professional knowledge about Education for Sustainability (EduS) both in theory and in practice in in-service teacher education contexts.

The projects’ goals are realized through three sequential but interrelated phases: (1) Construction of an EduS framework, emerging from a literature review and a characterization of teachers’ and teacher educators’ social representations; (2) design, implementation, and evaluation of teacher education courses, including action research projects for EduS in schools; (3) construction of a teacher education framework for EduS and dissemination of the project’s results at institutional, local, regional, national, and European levels.

The study presented in this chapter results from the work developed by the University of Aveiro team during the first phase of the project, which included the construction of a Framework for EduS purposes to guide the future actions of teachers and teacher educators. In the development of the Framework, researchers systematized and organized knowledge through a literature review of different types of documents that were considered to be relevant to developing teacher education for sustainability. These consisted of national education policy documents, Masters dissertations, and doctoral theses. This study focuses on the analysis conducted by the Aveiro team of doctoral theses in Education and aims to understand whether, how, and to what extent the key competencies in sustainability are present in these documents.

To address this objective, a qualitative study framed in an interpretative paradigm was conducted [16]. The study assumes a descriptive-interpretative strategy of an exploratory nature, aiming to identify the key competencies in education for sustainability (EduS) and to describe patterns and characteristics related to them in academic documents (doctoral theses) published in open-access national repositories. Considering the research objective, the literature review was used as a preferential research method to elicit meaning, gain understanding, and develop empirical knowledge.

### 2.2. Corpus of Analysis and Data Collection Procedures

The selection criteria for the academic documents considered for analysis were discussed at different national and transnational meetings of the TEDS project and considered the educational context of each country in the consortium. For the selection of academic documents, the Portuguese team privileged the scientific quality of the work, the scope of the research sources, the timeliness of the production, and the relevance of keywords. The documents were doctoral theses published in the Open-Access Scientific Repository of Portugal (RCAAP, https://www.rcaap.pt/, accessed on 12 February 2020), in the 2015–2020 timeframe, and in the Institutional Repository of the University of Aveiro (RIA, https://ria.ua.pt, accessed on 12 February 2020), in the 2010–2020 period. To conduct the search, the team used the following terms in Portuguese as well as their English translation: “educação para o desenvolvimento sustentável” (education for sustainable development), “educação para a sustentabilidade” (education for sustainability); “formação de professores” (teacher education). The term “formação de professores” (teacher education) was considered in full, while the remaining terms were used also in combination “educação AND desenvolvimento sustentável” (education AND sustainable development) and “educação AND sustentabilidade” (education AND sustainability) through Boolean operators. Title, abstract, or author-specified keywords were searched for instances of the terms.

The retrieved documents from both repositories are presented in Table 2.

### 2.3. Data Analysis Procedures

Deductive content analysis of the retrieved documents was carried out by the members of the Portuguese team of the TEDS project, based on the aforementioned framework, using the five key competencies in sustainability as categories of analysis.

The content analysis process followed the steps and procedures designed by Bardin [31], namely:

1. A pre-analysis to understand the documents’ structure and organization, which consisted of a floating reading of their content;
2. Text selection operations to code the excerpts according to the predefined categories;
3. A more detailed exploration of all the data, in which texts were divided into units of meaning and a code was assigned to each of these units [31,32].

The analysis was performed using WebQDA software (www.webqda.net, accessed on 23 August 2021) and proceeded as follows: Each researcher read through the documents and selected relevant passages, coding them as instances of a category and, within this, of a subcategory. Validation of the categorization process was performed by the members of the team organized in five groups. Each group was responsible for validating one of the categories and respective subcategories. In some cases, these meant that the excerpts were decoded and/or re-coded. The validation process allowed the standardization of the criteria used for the analysis. Once this stage was completed, the results were systematized.

Frequency counts of the relevant categories and subcategories were obtained, as well as of the number of coding references in each document. This provided a crude overall picture of the material being reviewed. Finally, the researchers proceeded with the interpretation of content, according to the project’s goals, selecting excerpts that translated the different competencies to understand whether, how, and to what extent the key competencies in sustainability were present in the doctoral theses.

Along the validation stage, some limitations and/or difficulties related to the categorization process were identified, namely:

1. Disparities regarding the understanding of categories of analysis by the team members, mainly due to different experiences and backgrounds, which eventually led to excerpts being categorized differently;
2. Inconsistency regarding the type of registration units (e.g., word, sentence, paragraph). For instance, some excerpts were very short, almost without clear meaning, and others were very long and dense, with no clear highlight of the component that had justified the researcher’s decision;
3. Conflict about the type of analysis to be carried out. The members of the team discussed the use of content analysis versus document analysis, opting for content analysis with discourse analysis outlines;
4. Resistance, by some members of the team, in using the WebQDA software.

## 3. Results

As previously mentioned, the analysis aimed to explore the understanding of EduS conveyed by the documents produced in Portugal (14 doctoral theses). Therefore, this analysis allowed us to perceive the way in which the key competencies in sustainability are present and are understood in the analyzed documents (i.e., areas to which they refer, their degree of explanation, or their attributed meanings).

Table 3 presents a systematization of the frequency counts of each category (i.e., key competencies in sustainability) per document.

As can be observed, all of the key competencies in sustainability considered in the analysis framework are present in the corpus, although not all of them were identified in all doctoral theses. Strategic Competency, with 182 references, was the competency with the highest number of references, followed by Anticipatory Competency, with 115. The competency with the fewest references in the corpus was Systems-Thinking Competency, with 46 references in total.

A more detailed presentation of the results considering the way in which each of these competencies is understood in the analyzed documents is presented below, organized by key competency.

### 3.1. Systems-Thinking Competency

Systems-Thinking Competency was identified in 12 of the 14 doctoral theses analyzed. All four subcategories are represented in different documents of the corpus.

The subcategory “Recognise and understand relationships” was mentioned the most, with 26 references. In the analysis, this subcategory is related to several types of interactions, such as interactions between different systems—“... to understand not only the interactions between human systems and physical, chemical, biological and geological systems, but also how human activity interacts and globally influences the environment, putting human survival at risk” (PhD_MM_2010) [23]—and interactions between different sustainable development dimensions—“... the importance of understanding the interconnection between economic, social and environmental problems is highlighted by the teachers involved in the study” (PhD_CCruz_2013) [25].

In general, within the analyzed theses, the recognition and understanding of relationships seem to be related to the multidimensionality of contemporary problems and their cause and effect. The subcategory “Analyse complex systems” is generally related to the examination of the complexity of political, social, economic, and cultural systems and of individual’s responsibility in the current planetary situation. This idea is explicitly presented in Costa (PhD_CC_2013) [26], which mentions the need to foster an “understanding of the problems we face and raising awareness of human responsibility in the current planetary situation, by promoting the development of proactive citizenships towards future change”.

Several documents in the corpus (16 references in 7 out of the 14 doctoral theses) stress the importance of learning how to deal with rapid transformation and uncertainty, which characterize the current world context. The subcategory “Deal with uncertainty” is also related to making responsible decisions and solving problems, in order to respond to the arising challenges. According to the documents, the process of cognitively apprehending the systemic dimension implies the development of an ability not only to understand how to deal with uncertainty and complexity but also to act. In other words, dealing with uncertainty demands being able to respond to challenges, to make responsible decisions, and to solve problems, as quoted: “Learning to live and deal with planetary emergency, in contexts of rapid and intense change, constitutes one of the most important challenges to education” (PhD_SS_2012) [24].

Systems-Thinking Competency is also intimately associated with critical thinking, as often stated by the literature. For instance, the documents (and their authors) emphasize that it is important to educate individuals to identify and understand relationships between people and things in the world, to analyze complex systems, and to understand the interdependence between systems, problems, and people, the problems that must be solved to build a more sustainable world, and the necessary domains of thinking and action.

### 3.2. Anticipatory Competency

In several of the doctoral theses analyzed, it was possible to find evidence of the category “Anticipatory Competency”, which involves specific analysis skills that focus on possible future trajectories and scenarios. Data analysis permitted the identification of 115 references distributed amongst the five subcategories around which this category is organized.

The subcategory “Understand and evaluate several futures” was the most represented, with a total of 51 references. In the theses, this subcategory is associated with the importance of preparing the next generations to understand the current planetary situation, the problems we face, and the challenges we will have to face in the future. This is evident in the following excerpt:

“... development is under permanent construction and it only makes sense if we think about the medium and long-term future, even if it requires immediate projection and action. Policies that meet sustainable development must consider education that is appropriate to the existing reality, to the projected and necessary, depending on the society that is intended to be reached”.

(PhD_MP_2016) [19]

The idea of a conscious intervention, based on values and oriented towards the improvement of future possibilities, is strongly shown in many references of this subcategory, which highlight the need to:

“enhance the understanding of the ethical dimensions of current problems, namely with regards to individual and collective actions and duties, in the sense of formulating opinions and acting with a scientific basis, analysing different alternatives”.

(PhD_CC_2013) [26]

The subcategory “Apply the principle of precaution” arose mostly related to ideas such as the importance of discovering “new ways of being with others and nature” and “discovering new ways of living with more wisdom and knowledge of the past” (PhD_SS_2012) [24]; the need to “change the paradigm so that current and future generations can live healthier lives, in harmony with ecosystems and in safety”; the importance of “educating for sustainability literacy to help students face complex problems” (PhD_AD_2015) [18]; and the “creation of protected areas in order to preserve the natural and cultural heritage” (PhD_JC_2014) [27].

The subcategory “Assess the consequences of actions” emerged closely associated with the idea of “learning from the past, not repeating experiences of discrimination, violence, persecution, exploitation,...” (PhD_SS_2012) [24]; the importance of making students aware of the rights and duties and responsibility of human beings in the current planetary situation (PhD_MP_2015, PhD_CC_2013) [17,26]; and the relevance of “evaluating alternative points of view reflecting on the importance of present decisions in the future and their consequences in different regions” (PhD_VC_2015) [30].

The subcategory “Deal with risk and change” was also represented in the corpus. This subcategory appears to be explicitly related to “the need to educate citizens who are aware of the seriousness and global nature of current issues” and the importance of “preparing citizens to live in a risk society, marked by unpredictability and complexity” (PhD_MP_2015) [17]. The same idea appears in different documents, presented in a similar way, for example, the “importance of each one’s commitment to an uncertain turnaround time” (PhD_MP_2016) [19] or even the “need to act effectively in a rapidly changing world” (PhD_CC_2013) [26].

It also highlighted the need to educate individuals who can select, analyze, and critically envision possibilities for the future, confronting and evaluating alternative points of view on actions for transforming the world. In practical terms, this means educating to anticipate alternative scenarios, based on concrete cases, simulations, and informed debates, reflecting the importance of conscious decision-making for the future of places, regions, and the world, combining both global and local scales of action (PhD_VC_2015) [30].

Overall, the doctoral theses highlight the need for this competency to be developed at cognitive, social, and emotional levels, and be presented transversally, in flexible curriculum management modalities. Therefore, the documents do not seem to lend the necessary weight—at least explicitly—to education’s role in helping individuals deal with risk and uncertainty.

### 3.3. Normative Competency

Normative competency appears in 9 out of the 14 doctoral theses in the corpus, with its two subcategories evidenced in different documents. This competency implies values-thinking and can be translated into thinking and acting with respect for nature and sustainability, contributing to equity and social justice.

In the doctoral theses analyzed, the subcategory “Understand and reflect on the norms and values that underlie people’s actions“ emerged mostly in relation to the valorization of an education oriented towards the preservation of linguistic and cultural diversity and based on human rights, as emphasized below:

“... educating for linguistic and cultural diversity, regarded as a way of educating for sustainability, is necessarily articulated with the purposes of an education for human rights, since this type of approach, by allowing students to contact with the richness of diversity, not only enables the development of the ability to communicate with people from different cultures, but also the reconstruction of their own identities and the construction of a sense of belonging to humanity”.

(PhD_SS_2012) [24]

The references coded in this subcategory also value an education “in which different values, perspectives and ideas are encouraged and treated respectfully, thereby allowing, space for deep conversation, creativity and innovation” (PhD_AD_2015) [18].

The documents also seem to value an autonomous school, capable of educating through flexible curriculum management, for environmental rights and duties, for human rights, diversity, participatory citizenship, and democracy, for literacy and critical thinking.

The subcategory “Negotiate sustainability values, principles, goals and targets” emerges from the corpus related to the valorization of an education for sustainability that “... helps citizens clarify the values and principles that guide them and change the idea they have of themselves in order to better collaborate in finding solutions to solve problems that affect the world” (PhD_MM_2010) [23]. In several documents, it is possible to find the idea that it is important “... to assure citizens the necessary knowledge for a full and active participation in the resolution of common problems...” (PhD_MP_2016) [19].

The documents also underline the importance of a critical stance towards oneself and towards others, “in which different values, perspectives and ideas are encouraged and treated respectfully, allowing thereby space for deep conversation, creativity and innovation” (PhD_AD_2015) [18].

In short, in order to build more sustainable societies, the literature [33] recommends promoting, in educational contexts, values such as diversity, equality, integration/inclusion, respect, democracy, participation, justice, and solidarity.

### 3.4. Strategic Competency

Strategic Competency appeared in all of the documents analyzed, with a total of 186 coded references. This competency is associated with action and education for social intervention, at different levels and educational contexts, in order to prepare individuals for the resolution of complex problems. This is visible, for instance, in the following statement, which urges “The academy, in general, including students and non-teaching staff (...) to seek and experiment new paths towards a culture of participation that allows broadening new ideas about creating sustainable universities” (PhD_AD_2015) [18].

Still, concerns related to sustainability are not restricted to environmental issues. Even though the most frequent keywords in the documents are “development”, “education”, and “sustainable”, all dimensions of sustainable development (society, economy, and environment) are expressed in the corpus. A possible interpretation for this might lie in the fact that the Brundtland Report [34], which presents what is considered to be the first definition of sustainable development, puts greater emphasis on the environmental issue.

Furthermore, EduS does not seem to be limited to the acquisition of knowledge and know-how but is rather seen as articulated with education for global citizenship, which values competencies for the exercise of active and responsible citizenship: “Education must, therefore, contemplate devices aimed at learning that amplify the knowledge of the world, societies, science, cultures, people. It is important that the information makes sense for the general population, that they can use it and benefit from it. An Education for Global Citizenship will certainly contribute to the education of more enlightened, informed and aware citizens about the problems that affect the territory” (PhD_MP_2016) [19], thus contributing to sustainable development, which “helps individuals understand existing social inequalities and act in a committed way seeking to transform society into a more just one” ([35], p. 2).

The documents also emphasize the need to renew and innovate educational practices, suitable for 21st-century society, involving active learning where students can link knowledge to their own experiences, and develop skills that allow them to be critically informed and engaged citizens. Several documents value “... learning in a real context, which provides skills for active citizenship and has the potential to generate effective and affective involvement in the community” (PhD_MP_2015) [17].

### 3.5. Interpersonal Competency

Interpersonal Competency is recognized as one of the key competencies for change agents working to solve sustainability problems and advance sustainability worldwide, resting on communication, teamwork, and stakeholder engagement. This competency was present in 11 out of the 14 theses analyzed, with all of its subcategories represented. The most represented subcategory was “Facilitate collaboration and participation in problem solving”, with 48 references in 8 different theses.

The prevailing ideas related to this competency highlighted in the analyzed documents are collaborative work with families and community agents, respect for and valorization of children/young people’s cultural diversity, problem-solving and organization skills, and empathy.

Interpersonal Competency emerged linked to the defense of a curriculum for diversity and sustainability, stressing the need to educate for intercultural understanding and global citizenship, based on values, such as social justice, equity, and peace. Another idea that is often stressed in the documents is that of a necessary balance between interpersonal and people relations with the planet. This is visible in the excerpt below:

“It is necessary to educate for the understanding of the planetary emergency situation that we face, developing intervention skills and planetary citizenship that integrate informed decision making and the adoption of attitudes/values of respect towards each other and towards the planet” (PhD_CC_2013) [26].

## 4. Discussion and Conclusions

This study aimed to understand whether, how, and to what extent the key competencies in sustainability are present in doctoral theses in education developed in the Portuguese Higher-Education context. With these goals in mind, a literature review of the documents, supported by deductive content analysis, was conducted.

Results of the analysis show that the competencies considered in the reference framework are present in research on education for sustainability carried out in recent years in Portugal. Indeed, in all of the documents analyzed, references were found for all of the categories (i.e., sustainability competencies) considered. Despite being stressed as being of major relevance by several authors and international organizations, such as Wiek et al., UNESCO, and Bamber [9,13,33], Systems-Thinking and Normative Competencies were the least represented in the corpus, in opposition to Strategic and Anticipatory Competencies, which were the most prominent. This seems to suggest that the authors of these theses lend emphasis to the behavioral dimension of learning, which focuses on promoting individual’s abilities to act towards building fairer and more sustainable futures and to (re)imagine and bring to life solutions to the problems communities and societies face nowadays. Curiously, these are not the competencies that seem to be emphasized in education policy documents and curricula. Indeed, as shown in several studies that have aimed to identify the presence and relevance of sustainability or global citizenship competencies, there seems to be a tendency for official guiding policy documents in Portugal (and elsewhere) to focus on the cognitive dimension of learning, related to promoting knowledge about global issues or environmentally aware practices, rather than on fostering committed and engaged student participation towards building more inclusive and sustainable societies [36,37].

Results also show that the understanding of the key competencies in sustainability that emerges from the analysis of the theses is coherent with the understanding that is proposed by UNESCO [13]. This agreement can be seen by comparing the description of each category (and subcategory) and the identified references (not just those selected for the text). Perhaps, hereafter, it would be interesting to present a brief summary of what was possible to determine concerning the understanding of each of the key competencies, as well as the strategies, resources, and approaches suggested by the authors to integrate these competencies in the school curriculum and in teacher education programs.

Finally, it should be stressed that, although it is possible to find similar understandings of these competencies in the theses, none of the authors make explicit reference to them, nor do they aim to define, identify, analyze, or promote this reference framework. This seems to suggest that these key competencies emerge in the investigation, despite not having been formally considered by the authors/researchers, also signaling that for the authors, the framework is still relatively unknown.

This study is not without limitations. One shortcoming is related to the size of the corpus, which consisted of 14 doctoral theses. Although this is not unusual in qualitative research, the small size of the corpus and its contextual nature (theses published in Portugal) make it impossible to generalize our results. Secondly, as highlighted in the methodology section, data analysis was performed by multiple researchers who might have had different understandings of the categories and subcategories of analysis. This factor, together with the subjectivity and positionality of the researchers when categorizing the data, might have influenced data analysis and our results. A possible solution for this would have been to conduct interviews with the authors of the theses to corroborate our findings. Unfortunately, due to the timeline of the project, this was considered impracticable. Despite these limitations, our findings underline the importance of conducting more research on the key competencies for sustainability, specifically through analyzing texts that describe intervention projects and suggest pedagogical pathways in integrating these competencies in the curriculum. Moreover, it seems fundamental to educate teachers about these competencies, namely by investing in pre-service and in-service teacher education that allows teachers to integrate these competencies in their teaching and professional knowledge. This might contribute to closing the gap between policy and practice in education for sustainability, which is one of the stumbling blocks in advancing progress towards achieving the SDGs.

## Figures and Tables

**Table 1:** Sustainability competencies and their analysis features.

Sustainability Competency	Analysis Features	
Systems-thinking competency	A	Recognize and understand relationships
	B	Analyze complex systems
	C	Think about how systems are embedded within different domains and different scales
	D	Deal with uncertainty
Anticipatory competency	A	Understand and evaluate several futures (possible, probable, and desirable
	B	Create one’s own visions of the future
	C	Apply the principle of precaution
	D	Assess the consequences of actions
	E	Deal with risk and change
Normative competency	A	Understand and reflect on the norms and values that underlie people’s actions
	B	Negotiate sustainability values, principles, goals and targets (in contexts of conflicts of interest and concessions)
Strategic competency	A	Collectively develop and implement innovative actions that promote sustainability (locally and in wider contexts)
Interpersonal competency	A	Be able to learn from others
	B	Understand and respect other people’s needs, perspectives and actions (empathy)
	C	Understand, relate to and be sensitive to others (empathic leadership)
	D	Handle group conflicts
	E	Facilitate collaboration and participation in problem solving

**Source:** Adapted from Juuti et al. ([15], p. 20).

**Table 2:** Corpus of analysis.

Title/Reference	Author	Year	Institution	Source	Code
Educação para a sustentabilidade: O uso de Sistemas de Informação Geográfica Participativos como instrumento de participação de crianças e adolescentes na construção de sociedades mais sustentáveis [17]	Preto, M.	2015	U. Minho	RCAAP	PhD_MP_2015
Participatory approaches in higher education’s sustainability practices: a mixed-methods study leading to a proposal of a new assessment model [18]	Disterheft, A.	2015	U. Aberta	RCAAP	PhD_AD_2015
Educação e desenvolvimento sustentável: Desafios na implementação de uma política pública intersetorial do Programa Mais Educação [19]	Pinheiro, M.D.	2016	U. Coimbra	RCAAP	PhD_MP_2016
Educação à distância e desenvolvimento local sustentável: as experiências de Brasil e Portugal [20]	Silva, K.	2016	U. Lisboa	RCAAP	PhD_KS_2016
Educação Ambiental para a Sustentabilidade: um estudo sobre a formação de futuros Licenciados em Biologia centrada no uso de aquários em projetos orientados para a ação ambiental sustentável no ensino médio [21]	Barreto, L.	2016	U. Minho	RCAAP	PhD_LB_2016
Sustainable Higher Education Institutions: Sustainable Development Challenges of Portuguese Higher Education Institutions [22]	Sousa, A.	2017	U. Aberta	RCAAP	PhD_AS_2017
Formação contínua de Professores de Ciências e de Filosofia-contributos de um estudo sobre educação para a sustentabilidade [23]	Morgado, M.	2010	U. Aveiro	RIA	PhD_MM_2010
Diversidade linguística e educação para um futuro sustentável [24]	Sá, S.	2012	U. Aveiro	RIA	PhD_SS_2012
A Educação para o Desenvolvimento Sustentável na formação de professores [25]	Cruz, C.	2013	U. Aveiro	RIA	PhD_CCruz_2013
Ciências no Primeiro Ciclo do Ensino Básico: um Programa para Educação para Desenvolvimento Sustentável [26]	Costa, M.C.	2013	U. Aveiro	RIA	PhD_CC_2013
Turismo sustentável e educação ambiental nos parques naturais de Montesinho e Douro Internacional [27]	Castro, J.	2014	U. Aveiro	RIA	PhD_JC_2014
Viver melhor na Terra: uma abordagem curricular para o 3.º CEB [28]	Macedo, C.	2015	U. Aveiro	RIA	PhD_CM_2015
Contributos da educação em Geociências para o desenvolvimento sustentável: uma abordagem ao tempo geológico [29]	Martins, L.	2015	U. Aveiro	RIA	PhD_LM_2015
Tecnologias de informação geográfica e promoção do pensamento espacial crítico: estratégias transdisciplinares em educação para o desenvolvimento sustentável no 3.º CEB [30]	Carlos, V.	2015	U. Aveiro	RIA	PhD_VC_2015

**Table 3:** Frequency counts of each category per document.

Matrix (E)	Systems-Thinking	Anticipatory	Normative	Strategic	Interpersonal
PhD_SS_2012	10	14	8	18	18
PhD_AS_2017	0	2	3	1	0
PhD_AD_2015	2	28	18	18	23
PhD_LB_2016	3	0	0	18	0
PhD_MP_2016	6	12	15	28	13
PhD_MP_2015	0	21	2	31	3
PhD_CM_2015	0	0	1	3	3
PhD_CC_2013	6	9	5	15	5
PhD_MM_2010	3	1	9	12	9
PhD_VC_2015	9	6	0	7	1
PhD_JC_2014	1	8	0	5	1
PhD_LM_2015	2	0	0	8	0
PhD_CCruz_2013	2	4	0	13	2
PhD_KS_2016	2	10	3	5	4
Total	46	115	64	182	82

## Data Availability

Not applicable.
